# Postoperative 20% albumin vs standard care and acute kidney injury after high-risk cardiac surgery (ALBICS): study protocol for a randomised trial

**DOI:** 10.1186/s13063-021-05519-8

**Published:** 2021-08-21

**Authors:** Mayurathan Balachandran, Piyusha Banneheke, Adrian Pakavakis, Wisam Al-Bassam, Vineet Sarode, Michael Rowland, Yahya Shehabi

**Affiliations:** 1 School of Clinical Sciences at Monash Health, Monash University, Clayton, Victoria Australia; 2 Intensive Care Services, School of Clinical Sciences at Monash Health, Monash University, Clayton, Victoria Australia; 3 Intensive Care Services, Cabrini Health, Malvern, Victoria Australia; 4 Cabrini Monash University Department of Medicine, Monash University, Malvern, Victoria Australia; 5 Department of Cardiothoracic Surgery, Barwon Health, Geelong, Victoria Australia; 6 University of New South Wales, Prince of Wales Clinical School of Medicine, Randwick, New South Wales Australia

**Keywords:** Albumin, Cardiac surgery, Acute kidney injury, Intensive care

## Abstract

**Background:**

Acute kidney injury (AKI) is a common complication of cardiac surgery. Factors such as cardiopulmonary bypass, aortic cross-clamping and surgical stress may precipitate renal hypoperfusion and ischaemia, inflammation and oxidative stress are associated with development of AKI. Albumin’s pharmacological properties and widespread availability have the potential to mitigate these factors. However, the effect of albumin on cardiac surgery-associated AKI is unknown.

**Objective:**

To evaluate the impact of postoperative 20% albumin infusion on kidney function after high-risk cardiac surgery.

**Methods:**

We designed an open-label, multicentre, randomised controlled trial—the ALBICS study (ALBumin Infusion and acute kidney injury following Cardiac Surgery). A total of 590 patients undergoing high-risk cardiac surgery (combined procedure or estimated glomerular filtration rate (eGFR) < 60 mL/min/1.73 m^2^) will be enrolled into the study and randomly allocated to receive a postoperative 20% albumin infusion or standard care in a 1:1 ratio, stratified by centre and baseline renal function. The study fluid will be administered upon arrival in intensive care for 15 h. Patients will be followed up until 28 days after surgery or until discharge from the hospital. The primary outcome is the proportion of patients who develop AKI in both groups. Secondary outcomes to be measured are proportions of AKI stage II and III, 28-day mortality, mechanical ventilation time and length of stay in intensive care and hospital.

**Conclusion:**

This trial aims to determine if a postoperative infusion of concentrated albumin reduces the risk of AKI following high-risk cardiac surgery.

**Trial registration:**

Australian New Zealand Clinical Trials Registry ACTRN12619001355167. Registered on 03 October 2019—retrospectively registered. https://www.anzctr.org.au/Trial/Registration/TrialReview.aspx?id=378383.

## Administrative information

The order of the items has been modified to group similar items (see http://www.equator-network.org/reporting-guidelines/spirit-2013-statement-defining-standard-protocol-items-for-clinical-trials/

**Table:** 

Title {1}	Postoperative 20% albumin vs standard care and acute kidney injury after high-risk cardiac surgery (ALBICS): study protocol for a randomised trial
Trial registration {2a and 2b}.	Australian New Zealand Clinical Trials Registry, 03 October 2019 (ACTRN1261900135516703)
Protocol version {3}	Version 2.11, 30 June 2021
Funding {4}	This study is internally funded by each hospital’s intensive care unit and/or cardiothoracic department.
Author details {5a}	Mayurathan Balachandran^1^, Piyusha Banneheke^1^, Adrian Pakavakis^2^, Wisam Al-Bassam^2^, Vineet Sarode^3,4^, Michael Rowland^5^, Yahya Shehabi^2,6^ 1. School of Clinical Sciences at Monash Health, Monash University, Clayton, Victoria, Australia 2. Intensive Care Services, School of Clinical Sciences at Monash Health, Monash University, Clayton, Victoria, Australia 3. Intensive Care Services, Cabrini Health, Malvern, Victoria, Australia 4. Cabrini Monash University Department of Medicine, Monash University, Malvern, Victoria, Australia 5. Department of Cardiothoracic Surgery, Barwon Health, Geelong, Victoria, Australia 6. University of New South Wales, Prince of Wales Clinical School of Medicine, Randwick, New South Wales, Australia
Name and contact information for the trial sponsor {5b}	School of Clinical Sciences at Monash HealthContact: Prof Yahya ShehabiDirector of ICU Researchyahya.shehabi@monashhealth.org
Role of sponsor {5c}	This is an investigator initiated study. The study sponsor is the clinical school where the chief investigator is employed. The clinical school provides administrative, logistic and other supports that are required for this study.

## Background and rationale {6a}

Acute kidney injury is a well-recognised complication of cardiac surgery (CSA-AKI). A large retrospective cohort study of 25,086 patients reported CSA-AKI in 30% of patients [1]. This study also demonstrated odds ratios of in-hospital mortality equal to 3.17 in Acute Kidney Injury Network (AKIN) stage 1 and 43.77 in AKIN stage 3. Given that over one million patients undergo cardiac surgery each year, CSA-AKI presents a significant burden of disease [2].

### Pathophysiology and risk factors

**Fig. 1:**
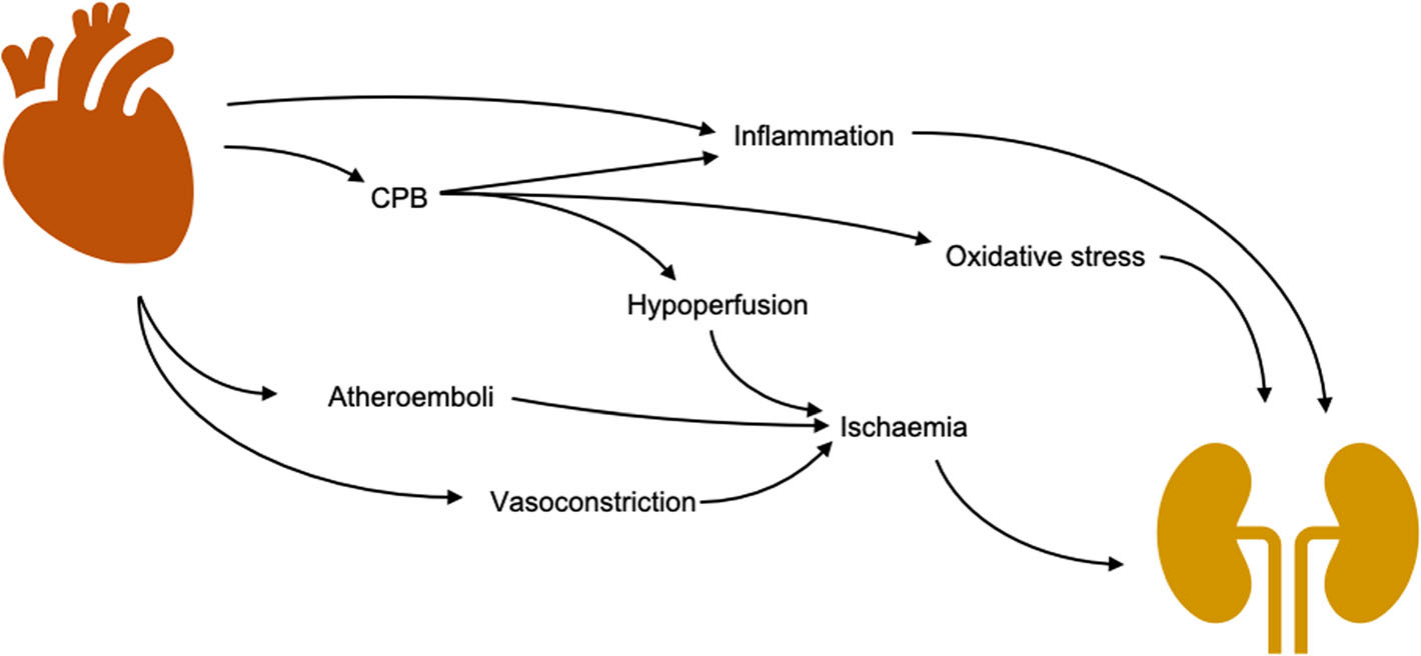
Pathophysiology of CSA-AKI

Current understanding of the pathophysiological mechanisms surrounding CSA-AKI are limited. There is unlikely to be a single mechanism responsible for AKI in these patients but instead a myriad of injurious pathways that take place in the perioperative period. Many of these pathways relate to renal perfusion, inflammation and oxidative stress (Fig. 1).

Cardiopulmonary bypass (CPB) is associated with postoperative renal injury, likely via a reduction in renal blood flow and glomerular filtration [3]. Non-pulsatile flow may also result in an imbalance between cortical and medullary perfusion [4]. Haemodilution is an inevitable consequence of CPB and has been identified as an independent predictor of CSA-AKI [5–7]. The renal medulla and corticomedullary junction are particularly vulnerable to these changes given their relative hypoxia in comparison to other tissues [8, 9]. Prolonged hypoperfusion precipitates ischaemic changes such as inflammatory cell infiltration, cell contraction and necrosis [10]. Other operative risk factors include aortic cross-clamp time, blood transfusion, inotrope requirements and the complexity of the procedure performed [11, 12].

Cardiac surgery results in increased levels of pro-inflammatory cytokines, notably interleukin (IL)-6 and tumour necrosis factor (TNF)-α [13] with increased risk of AKI after cardiac surgery [14–16]. Subsequent activation of macrophages, neutrophils and lymphocytes lead to renal parenchymal infiltration and development of AKI [17, 18].

### Hypoalbuminaemia and cardiac surgery

Hypoalbuminaemia is common after cardiac surgery. A large observational study (*n* = 2818) found that 94% of patients with normal preoperative serum albumin levels developed hypoalbuminaemia at 24 h after cardiac surgery, where the risk of AKI approximately doubled with each 5 g/l reduction [19].

In separate meta-analyses of 90 cohort studies and 9 RCTs totalling 291,433 and 535 acutely ill patients, Vincent et al. found that a 10 g/l reduction in serum albumin correlated with an increased odds of mortality and morbidity of 116% and 52% after cardiac surgery [20]. The analysis of randomised trials also suggests that albumin supplementation to maintain serum levels greater than 30 g/l decreases the rate of complications. These results contend that hypoalbuminaemia is common after cardiac surgery and its correction may prevent AKI.

Albumin administration may not be useful in all critically ill populations. A recently published randomised trial of 828 hypoalbuminaemic patients with acute complications of decompensated cirrhosis randomised patients to receive daily 20% albumin infusions titrated to the degree of hypoalbuminaemia [21]. Despite increasing serum albumin concentrations, albumin administration failed to improve outcomes with respect to in-hospital kidney dysfunction and mortality at 28 days, 3 months and 6 months.

### Nephroprotective properties of albumin

Albumin may have nephroprotective effects via different mechanisms. Albumin infusion reduces fluid overload which is an independent risk factor for AKI in critically ill patients [22–24]. A rise in venous pressure and subsequent renal interstitial oedema impair glomerular filtration [25] and may reduce cortical oxygen pressures [26].

The HAS FLAIR trial found similar results comparing 20% albumin and crystalloid fluid bolus therapy over the first 24 h after cardiac surgery (median 1100 ml vs 1970 ml, *p* = 0.001, *n* = 100) [27]. Additionally, the SWIPE trial found that in a sample of 321 ICU patients, half of whom had undergone cardiac surgery, 20–25% albumin preparations reduced fluid requirements in the first 48 h compared to 4–5% albumin preparations (median 3429 ml vs 4217 ml, *p* = 0.06) [28].

Injury to the endothelium contributes to the development of AKI. Endothelial damage increases leukocyte adherence, platelet aggregation, vasoconstriction and podocyte dysfunction [29]. Loss of the glycocalyx is thought to relate to microthrombi, ischaemia reperfusion injury, oxidative stress and systemic inflammation; all of which may occur following CPB [10, 30, 31]. Administration of albumin protects the glycocalyx. In animal models, administration of albumin resulted in reduced measures of glycocalyx dysfunction [32, 33].

Albumin contributes to antioxidant activity in plasma. Over 70% of free radical neutralisation is attributable to albumin [34]. This property is predominantly due to its abundance and the existence of a thiol group in the cysteine-34 position of its molecular structure. Thiols are known for their reaction with, and ‘trapping’ of, reactive oxygen species. In healthy subjects, 70–80% of serum albumin is reduced at the cysteine-34 thiol, while an additional 25% may be oxidised further [35, 36].

### Clinical trials of albumin and cardiac surgery

In the first and only clinical trial investigating albumin in this way, Lee et al. randomised 220 patients with undergoing off-pump cardiac surgery to receive either preoperative 20% albumin or saline [37]. This double-blind study included patients with preoperative serum albumin level less than 40 g/l. The volume of fluid administered varied between 100 and 300 ml and was titrated to each patient’s preoperative serum albumin level. Albeit limitations, the trial reported reduced risk of AKI by Kidney Disease: Improving Global Outcomes (KDIGO) [38] (RR = 0.533, *p* = 0.048) and Acute Kidney Injury Network (AKIN) criteria (RR = 0.557, *p* = 0.031) with albumin administration.

There is insufficient scientific literature to determine the utility of exogenous albumin to prevent CSA-AKI. More clinical trials are needed in this area.

## Objectives {7}

We hypothesise that an infusion of 20% albumin will reduce the proportion of patients who develop postoperative AKI after high-risk cardiac surgery, when compared to standard care.

The objective of this study is to evaluate the impact of postoperative 20% albumin infusion on kidney function after high-risk cardiac surgery.

## Trial design {8}

The ALBICS (ALBumin Infusion and acute kidney injury following Cardiac Surgery) study is a multicentre, parallel-group, open-label, prospective, randomised controlled, superiority trial.

### Methods: participants, interventions and outcomes

#### Study setting {9}

Patients will be recruited at the following Australian hospitals: Monash Medical Centre, Cabrini Hospital, University Hospital Geelong, Prince of Wales Hospital, Prince of Wales Private Hospital and the Austin Hospital.

#### Eligibility criteria {10}

To be included in the study, patients must meet all of the following inclusion criteria:

- Aged 18 years or older;
- Have undergone cardiac surgery;
- At least one of the following:
  i. Estimated glomerular filtration rate (eGFR) < 60 mL/min/1.73 m^2^, or
  ii. Have had a combined valve and coronary procedure, or
  iii. Two or more valve procedures, or
  iv. Surgery involving the thoracic aorta.

Patients meeting any of the following exclusion criteria will be excluded from the study:

- eGFR < 15 mL/min/1.73 m^2^,
- Serum albumin < 20 g/l,
- Dialysis dependence,
- Kidney transplant,
- Undergone off-pump cardiac surgery,
- Requiring extra-corporeal life support or ventricular assist device immediately postoperative,
- Jehovah’s Witness.

#### Who will take informed consent? {26a}

A study investigator will obtain informed consent from each participant prior to enrolment in this study. The investigator will discuss the risks and benefits of participation and will check that the patient comprehends the information provided. Consent will be voluntary and free from coercion.

#### Additional consent provisions for collection and use of participant data and biological specimens {26b}

Not applicable.

## Interventions

### Explanation for the choice of comparators {6b}

Patients will be randomly allocated to either the intervention group (albumin 20% and standard care) or the comparator group (standard care only). Standard care was chosen as an appropriate comparator given that the intervention is proposed as an adjunct to routine care.

### Intervention description {11a}

Patients randomised to the intervention arm will receive an intravenous infusion of 20% albumin for 15 h at 20 ml/h as soon as possible after ICU admission, where the time to initiation of infusion does not exceed 6 h.

Patients randomised to the standard care arm will not receive any 20% albumin for the first 24 h of ICU admission.

### Relevant concomitant care permitted or prohibited during the trial {11d}

Both treatment groups will receive standard care as per the clinician in charge. This includes any background treatments considered routine care such as vasopressors, inotropes, ventilation and initiation of dialysis. Administration of 4% albumin will not be restricted.

### Criteria for discontinuing or modifying allocated interventions {11b}

For those randomised to receive 20% albumin, the infusion may be ceased if the patient develops an allergic reaction or fluid overload (shortness of breath, increased oxygen requirements).

For those randomised to receive standard care, 20% albumin may be administered within the first 24 h if it is deemed clinically indicated. These decisions will be at the discretion of the clinician in charge.

### Strategies to improve adherence to interventions {11c}

The design for the ALBICS study will be presented to staff at each participating site, with input from intensive care specialists, cardiac surgeons and cardiac anaesthetists. These meetings will improve consistency of protocol implementation. Other measures include presentations and visual information provided to clinical staff to reduce the risk of inadvertent protocol deviation.

### Provisions for post-trial care {30}

All patients enrolled in this trial will receive standard postoperative surgical care beyond 24 h. Patients will be followed as per the study protocol.

### Outcomes {12}

The primary outcome is the proportion of patients that develop AKI within hospital stay up to Day 28 post-enrolment. AKI is defined by creatinine-based KDIGO criteria (Table 1 Pathophysiology of CSA-AKI) [38]. Patients after cardiac surgery often have polyuria in the first few hours after surgery and they are frequently given diuretic therapy after day 1 postoperatively. For this reason, urine output may not a be a reliable measure of AKI.

**Table 1:** KDIGO creatinine-based criteria for diagnosis and staging of AKI

Stage	Serum creatinine
I	1.5–1.9 times baseline within 7 days; or ≥ 26.5 μmol/l (≥ 0.3 mg/dl) increase within 48 h
II	2.0–2.9 times baseline within 7 days
III	≥ 3.0 times baseline within 7 days; or Increase to ≥ 353.6 μmol/l (≥ 40 mg/dl); or Initiation of renal replacement therapy

Secondary and tertiary outcomes are described in Table 2. These outcomes were chosen as appropriate measures of acute deterioration or recovery, and the haemodynamic effects of albumin.

**Table 2:** Study outcomes

Primary outcome	In-hospital AKI Stage I, II or III (proportion of patients)
Secondary outcomes	In-hospital AKI stage II and III (proportion of patients) In-hospital mortality ICU LOS (hours) Hospital LOS (days) Ventilation time (hours)
Tertiary outcomes	Peak SCr (μmol/l) Serum albumin at 24 h (g/l) Serum Hb at ICU discharge (g/l) Proportion of patients needing inotrope and/or vasopressor therapy Net fluid balance at 48 h (ml) Quantity of RBCs transfused over first 48 h (ml) Initiation of CRRT

*AKI*, acute kidney injury; *CRRT*, continuous renal replacement therapy; *Hb*, haemoglobin; *ICU*, intensive care unit; *LOS*, length of stay; *RBC*, red blood cell; *SCr*, serum creatinine

### Participant timeline {13}

**Fig. 2:**
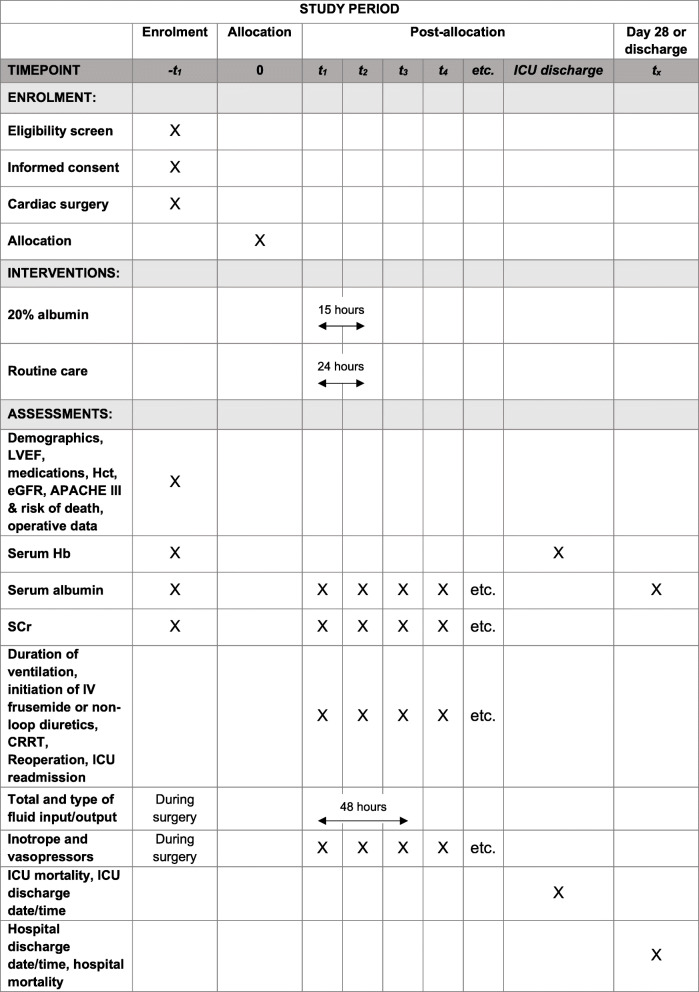
Data collection timeline (SPIRIT figure). CRRT, continuous renal replacement therapy; eGFR, estimated glomerular filtration rate; Hb, haemoglobin; Hct, haematocrit; ICU, intensive care unit; LVEF, left ventricular ejection fraction; SCr, serum creatinine

Patients will be followed up until the 28th day post-enrolment or hospital discharge, whichever occurs first. Data collected will be restricted to the parameters that are necessary to define clinical characteristics and will be obtained from routine laboratory investigations and hospital records (Fig. 2). This includes baseline demographics, outcome measures, physiological parameters, operative data, therapeutic interventions, death and other adverse events.

### Sample size {14}

The sample size for the study was determined by a priori power analysis. The anticipated incidence of AKI in the comparator group was based upon a cohort of over 25,000 patients that reported AKI in 30% of patients undergoing cardiac surgery [1]. Based on this estimate, the inclusion of 590 patients will achieve 80% power to detect a 10% absolute risk reduction in the risk of AKI (2-sided *p* = 0.05).

### Recruitment {15}

Recruitment will take place at metropolitan teaching hospitals that see a large number of cardiac cases. This will allow sufficient enrolment to reach the target sample size in a timely manner.

## Assignment of interventions

### Sequence generation {16a}

A permuted block, computer-generated, randomisation sequence with fixed block size, stratified by hospital and eGFR (< 60 mL/min/1.73 m^2^ or > 60 mL/min/1.73 m^2^) will be used to allocate participants in a 1:1 ratio.

### Concealment mechanism {16b}

Patients will be allocated using REDCap (Research Electronic Data Capture), a computer-based software for data collection. REDCap will use the prepared randomisation schedule to determine the allocation of the participant in a traceable manner, such that the treatment group is not knowable prior to allocation and cannot be changed after it. While blinding clinicians to treatment allocation is desirable, it is not deemed feasible for this study. We minimise bias through allocation concealment and evaluation of objective laboratory-based data.

### Implementation {16c}

The allocation sequence will be generated by a person not involved with enrolment or future analysis. Participants will be screened and randomised on admission to intensive care by a site investigator or member of research support staff.

### Blinding {17a}

While blinding is a desirable feature of clinical trials, the study outcomes are distant from the intervention and the open-label design is unlikely to influence the outcome at 28 days.

### Procedure for unblinding {17b}

Not applicable.

## Data collection and management

### Plans for assessment and collection of outcomes {18a}

All study data will be collected by research staff at each site using an electronic case report form and stored in a password-protected, traceable, database managed by Monash University. All parameters are defined in a data dictionary detailing the way in which data should be collected. The central coordinating investigators will ensure site visits for data monitoring, timely resolution of queries and correction of errata during quality control checks.

### Plans to promote participant retention and complete follow-up {18b}

The intervention is to be administered soon after ICU admission and will last a short duration of time, likely while participants are unconscious. Patients will be approached once conscious and practical to do so, to inform them of study progress and follow-up required. Obtaining data from medical records and laboratory data will ensure complete follow-up.

### Data management {19}

Study data was collected and managed using REDCap electronic data capture tool hosted and managed by Helix (Monash University) [39, 40]. REDCap is a secure, web-based software platform designed to support data capture for research studies, providing [1] an intuitive interface for validated data capture [2]; audit trails for tracking data manipulation and export procedures [3]; automated export procedures for seamless data downloads to common statistical packages; and [4] procedures for data integration and interoperability with external sources.

### Confidentiality {27}

Wherever possible, identifying information will be removed. Only de-identified data will be entered into the case report form. Identifying documents such as consent forms will be kept in locked rooms that may only be accessed by authorised personnel.

### Plans for collection, laboratory evaluation and storage of biological specimens for genetic or molecular analysis in this trial/future use {33}

Not applicable. This study evaluates data collected via routine laboratory investigation.

## Statistical methods

### Statistical methods for primary and secondary outcomes {20a}

Normality of continuous variables will be assessed and log-transformed where appropriate. Equality of variance for normally distributed variables will be assessed using Levene’s test. Between-group comparisons will be performed using chi-squared tests for equal proportion, Student’s *t* test for continuous variables with equal variance, Welch’s *t* test for continuous variables with unequal variance, and Mann-Whitney *U* test otherwise. Categorical variables will be described as frequency with proportions (%). Continuous variables will be expressed as mean ± standard deviation if normally distributed, and median with interquartile range if not normally distributed. Analysis will be performed using R version 4.0.2 (R Foundation for Statistical Computing, Vienna, Austria).

### Methods for additional analyses {20b}

A subgroup analysis will be performed in patients with and without baseline renal insufficiency (eGFR < 60 mL/min/1.73 m^2^ or ≥ 60 mL/min/1.73 m^2^).

### Interim analyses {21b}

An interim analysis is not planned.

### Methods in analysis to handle protocol non-adherence and any statistical methods to handle missing data {20c}

Data will be analysed using an intention-to-treat methodology. A per-protocol sensitivity analysis will also be conducted. Ongoing site education would reduce the risk of protocol deviation. We will adjust for missing data using multiple imputation.

## Oversight and monitoring

### Composition of the coordinating centre and trial steering committee {5d}

The trial steering committee is composed of investigators from the departments of cardiothoracic surgery and intensive care. The chair of the steering committee, through the School of Clinical Sciences at Monash Health, will be coordinating this study.

### Composition of the data monitoring committee, its role and reporting structure {21a}

Albumin administration is considered part of routine postoperative care after cardiac surgery. In addition, the open-label design will allow the assignment of adverse events to the intervention. Serious adverse events will be reviewed regularly by the study steering committee and reported to the ethics committee.

### Adverse event reporting and harms {22}

Adverse events will be collected in accordance with the Monash Health Human Research Ethics Committee guidelines. Serious adverse reactions will be reported to the institutional research support services at each site. Significant safety issues will be reported to the Monash Health Human Research Ethics Committee.

### Frequency and plans for auditing trial conduct {23}

After each site is activated and has enrolled five patients, site monitoring of the consenting process, protocol adherence and data collection will be conducted. At the end of this study, all sites will be monitored for protocol adherence and completion of data collection.

### Plans for communicating important protocol amendments to relevant parties {25}

Protocol amendments will be promptly distributed to all relevant parties via the appropriate channels, for example by email or formal ethics review.

## Dissemination plans {31a}

The study will be published in the name of the individual investigators and in the group name ‘ALBICS study investigators’. Full credit will be given to all collaborating investigators, research staff and institutions. All authors will comply with the internationally agreed upon requirements for authorship and will approve the manuscript before submission.

The final results will be presented at one or more major scientific meetings and will be published in a peer-reviewed scientific journal that discusses care of critically ill patients. We will ensure that results are available to study participants and for translation to intensive care with concurrent recommendations for change in practice based on the study findings.

## Conclusions

Acute kidney injury after cardiac surgery is common. Cardiopulmonary bypass appears to be an important factor and may precipitate AKI through various mechanisms related to renal hypoperfusion, inflammation and oxidative stress. Albumin’s oncotic and pharmacological properties demonstrate its potential benefit for the prevention of CSA-AKI. A large, multicentre randomised trial is needed to determine this definitively. The ALBICS study is designed to answer this question.

### Trial status

Recruitment began on 08 July 2019. As at October 2020, 25 patients have been enrolled in the ALBICS study. The COVID-19 pandemic has had a significant impact on study progress, having halted recruitment at all participating sites. The study recommenced recruitment on 19 July 2021 and we expect the trial to reach completion by the end of 2023. Working protocol version 2.11, 30 June 2021. A protocol amendment was approved in July 2021.

## Data Availability

The datasets for the completed study will be available according to Monash University data sharing protocols. The authors have no contractual agreements to disclose that would limit such access.

## Abbreviations

AKI: Acute kidney injury
AKIN: Acute Kidney Injury Network
CPB: Cardiopulmonary bypass
CSA-AKI: Cardiac surgery-associated acute kidney injury
eGFR: Estimated glomerular filtration rate
ICU: Intensive care unit
IL: Interleukin
KDIGO: Kidney Disease: Improving Global Outcomes
mRNA: Messenger ribonucleic acid
REDCap: Research Electronic Data Capture
RCT: Randomised controlled trial
SBP: Spontaneous bacterial peritonitis
SPIRIT: Standard Protocol Items: Recommendations for Interventional Trials
TNF: Tumour necrosis factor
